# Development of a Cell-Based Assay Measuring the Activation of FcγRIIa for the Characterization of Therapeutic Monoclonal Antibodies

**DOI:** 10.1371/journal.pone.0095787

**Published:** 2014-04-21

**Authors:** Minoru Tada, Akiko Ishii-Watabe, Takuo Suzuki, Nana Kawasaki

**Affiliations:** Division of Biological Chemistry and Biologicals, National Institute of Health Sciences, Tokyo, Japan; Pasteur Institute of Shanghai, Chinese Academy of Science, China

## Abstract

Antibody-dependent cellular cytotoxicity (ADCC) is one of the important mechanisms of action of the targeting of tumor cells by therapeutic monoclonal antibodies (mAbs). Among the human Fcγ receptors (FcγRs), FcγRIIIa is well known as the only receptor expressed in natural killer (NK) cells, and it plays a pivotal role in ADCC by IgG1-subclass mAbs. In addition, the contributions of FcγRIIa to mAb-mediated cytotoxicity have been reported. FcγRIIa is expressed in myeloid effector cells including neutrophils and macrophages, and it is involved in the activation of these effector cells. However, the measurement of the cytotoxicity via FcγRIIa-expressing effector cells is complicated and inconvenient for the characterization of therapeutic mAbs. Here we report the development of a cell-based assay using a human FcγRIIa-expressing reporter cell line. The FcγRIIa reporter cell assay was able to estimate the activation of FcγRIIa by antigen-bound mAbs by a very simple method *in vitro*. The usefulness of this assay for evaluating the activity of mAbs with different abilities to activate FcγRIIa was confirmed by the examples including the comparison of the activity of the anti-CD20 mAb rituximab and its Fc-engineered variants, and two anti-EGFR mAbs with different IgG subclasses, cetuximab (IgG1) and panitumumab (IgG2). We also applied this assay to the characterization of a force-oxidized mAb, and we observed that oxidation significantly decreased the FcγRIIa activation by EGFR-bound cetuximab. These results suggest that our FcγRIIa reporter assay is a promising tool for the characterization of therapeutic mAbs, including Fc-engineered mAbs, IgG2-subclass mAbs, and their product-related variants.

## Introduction

Antibody-dependent cellular cytotoxicity (ADCC) plays an important role in anti-tumor activity of therapeutic monoclonal antibodies (mAbs) targeting tumor cells.[1]–[3] To date, many mAbs exhibiting ADCC activity have been approved and have contributed to anticancer therapy (e.g., the anti-EGFR (epidermal growth factor receptor) mAb cetuximab for colorectal cancers, the anti-CD20 mAb rituximab for B-cell lymphomas, and the anti-HER2 mAb trastuzumab for metastatic breast cancers). In addition, for the enhancement of ADCC activity, antibody Fc engineering by amino acid substitution [4]–[7] or glycoform modification [8]–[13] has been advanced. The glyco-engineered anti-CCR4 mAb mogamulizumab [14] and anti-CD20 mAb obinutuzumab [15] were recently approved in Japan and United States, respectively, and various Fc-engineered mAbs with higher ADCC activity are currently under development[16].

Activating Fcγ receptors (FcγRs) play a critical role in ADCC. mAbs bound to a cell-surface antigen interact with FcγRs expressed on effector cells such as natural killer (NK) cells, neutrophils and macrophages, inducing these cells to exert cytotoxicity. In humans, there are four types of activating FcγRs: FcγRI, FcγRIIa, FcγRIIIa and FcγRIIIb.[17] FcγRIIIa is the only activating FcγR expressed on NK cells, and it is thought to play a pivotal role in ADCC induced by IgG1 subclass mAbs. Its significance in clinical efficacy is supported by reports of correlations between FcγRIIIa polymorphism (F158V) and response to rituximab [18], [19] or other mAbs. Much of the Fc engineering in tumor-targeting mAbs has increased the affinity to FcγRIIIa, resulting in enhanced ADCC activity via NK cells.

To assess ADCC activity of these mAbs, various methods of assaying ADCC have been reported. In most cases, the killing of target cells is measured by using human peripheral mononuclear blood cells (PBMCs) from donated blood or isolated primary NK cells as effector cells. Although these assays can directly assess the cytotoxicity induced by mAbs via effector cells, they have several drawbacks in their implementation for routine testing, such as the requirement of fresh blood from donors and insufficient repeatability caused by the differences in effector cell activity among donors. To resolve these problems, alternative assays to measure the binding or activation of FcγRIIIa by antigen-bound mAbs have been developed. Miller et al. reported that an enzyme-linked immunosorbent assay (ELISA)-based bridging assay using recombinant FcγRIIIa protein can be used as a surrogate assay for ADCC activity.[20] Parekh et al. developed an ADCC-reporter gene assay measuring the activation of FcγRIIIa-expressing reporter cells with excellent performance in accuracy, precision and robustness.[21]

In addition to FcγRIIIa, the role of FcγRIIa in the efficacy of mAbs has been studied. Several clinical studies indicated the correlations between FcγRIIa polymorphism (R131H) and the response to IgG1 subclass mAbs such as rituximab [19] and cetuximab,[22] although the mechanism underlying these correlations is unclear because no significant difference was observed in the *in vitro* binding to human IgG1 between 131R and 131H alleles.[23] However, the importance of FcγRIIa in mAb-mediated cytotoxicity via immune effector cells other than NK cells has been reported. FcγRIIa is widely expressed in myeloid effector cells and plays a pivotal role in the activation of neutrophils [24]–[26] and macrophages.[6] Fc-engineered mAbs with higher FcγRIIa affinity by amino-acid substitutions have been developed, and their use succeeded in the enhancement of the mAb-mediated phagocytosis of tumor cells by macrophages [6].

In addition, FcγRIIa is a major receptor for IgG2 subclass mAbs. The IgG2-mediated elimination of infectious pathogens by myeloid effector cells plays an important role in protective immune responses. Thus, therapeutic IgG2-subclass mAbs may elicit effector functions via myeloid effector cells by FcγRIIa activation. Indeed, FcγRIIa was reported to be involved in the myeloid effector cell-mediated cytotoxicity by panitumumab, a human IgG2 mAb against EGFR.[27] Therefore, it is important to evaluate the mAb-dependent activation of FcγRIIa as well as that of FcγRIIIa in the development of tumor-targeting therapeutic mAbs of both the IgG1 and IgG2 subclasses. However, the main effector cells exerting ADCC in human PBMCs used for traditional ADCC assays are NK cells expressing FcγRIIIa, and these assays assess only the contribution of FcγRIIIa activation by mAbs. To assess the cytotoxicity via other effector cells expressing FcγRIIa, it is necessary to isolate primary neutrophils from fresh blood or to differentiate macrophages from primary monocytes and these processes may lead to variability of the assay.

The purpose of the present study was to establish a cell-based assay to conveniently measure mAb-dependent FcγRIIa activation. We developed an FcγRIIa-expressing reporter cell line in which the reporter luciferase gene expresses depending on the activation of FcγRIIa via crosslinking by antigen-bound mAbs. Cell-based assays using our reporter cell line are a promising tool for the assessment of Fc-engineered mAbs with different FcγRIIa-binding affinities or IgG2-subclass mAbs, and they would also be useful for the characterization of mAb product-related variants.

## Materials and Methods

### Cell Culture

Jurkat (RCB0806) cells were provided by the RIKEN BRC and cultured in RPMI1640 medium supplemented with 10% fetal bovine serum (FBS). Daudi (JCRB9071) and A431 (JCRB0004) cells were obtained from the JCRB cell bank. Daudi cells were cultured in RPMI1640 medium supplemented with 20% FBS. A431 cells were cultured in DMEM high glucose with GlutaMAX (Life Technologies) supplemented with 10% FBS and 1 mM sodium pyruvate.

### Establishment of the Jurkat/FcγR/NFAT-Luc Cell Line

We generated cDNA encoding human FcγRIIa/131H by an inverse polymerase chain reaction (PCR) method using cDNA encoding FcγRIIa/131R (Open Biosystems) as a template and subcloned into pVITRO1-neo-mcs vector (InvivoGen). We subcloned cDNA encoding human FcγRIIIa/158V (OriGene) and Fcγ chain (Open Biosystems) into pVITRO1-neo-mcs vector. Jurkat cells were transfected with pVITRO1-neo-FcγRIIa/131H or pVITRO1-neo-FcγRIIIa/158V+Fcγ chain by Nucleofector (Lonza).

Stable cell lines expressing FcγRIIa or both FcγRIIIa and Fcγ chain were screened by selection using 500 µg/mL G418 (Nacalai Tesque) and the limited dilution method, followed by a flow cytometric analysis to confirm the expression of FcγRs. To generate the cell line co-expressing NFAT-driven luciferase reporter gene, we transfected Jurkat/FcγRIIa and Jurkat/FcγRIIIa cells with pGL4.30[*luc2P*/NFAT-RE/Hygro] vector (Promega) containing hygromycin-resistance gene. We confirmed the activation of NFAT-driven luciferase reporter by conducting FcγR-crosslinking assays using anti-FcγR antibodies.

### Flow Cytometric Analysis

We analyzed the cell surface expression of FcγRs using the FACSCanto II flow cytometer (BD Biosciences, San Diego, CA) using fluoresceine-isothiocyanate (FITC)-conjugated anti-CD32 monoclonal antibody (clone FLI8.26, BD Biosciences) or anti-CD16 monoclonal antibody (clone 3G8, BD Biosciences). For the flow cytometry-based bridging assay, Daudi and Jurkat/FcγRs cells were labeled with Calcein AM (eBioscience) and Calcein Violet 450 AM (eBioscience) respectively according to the manufacturer’s instructions. Fluorescently-labeled Daudi (3×10^4^ cells/well) and Jurkat/FcγRs (3×10^5^ cells/well) were co-cultured in a 96-well plate for 30 min in the presence of 10 µg/mL rituximab or control human IgG1 (SIGMA), and then analyzed by the FACSCanto II flow cytometer.

### FcγR Crosslinking Assay

We performed the FcγR crosslinking assay as described.[28] Jurkat/FcγRs cells were washed with Opti-MEM I Reduced Serum Media (Life Technologies) and incubated on ice for 30 min with the medium containing 15 µg/mL mouse anti-FcγRs monoclonal antibody: anti-CD32 (clone IV.3, StemCell Technologies,) or anti-CD16 (clone 3G8, BD Biosciences). After being washed three times with the medium, the cells were suspended in the medium and warmed to 37°C for 10 min, and then crosslinked by goat F(ab’) 2 anti-mouse IgG (Beckman Coulter), followed by incubation at 37°C. At each time point, the cells were lysed by adding (2×) lysis buffer (100 mM Tris-HCl, pH 7.5, 300 mM NaCl, 2% Nonidet-P40, 0.5% deoxycholate, (2×) Protease Inhibitor Cocktail [Nacalai Tesque], and (2×) Phosphatase Inhibitor Cocktail [Nacalai Tesque]) and centrifuged at 20,000 g for 15 min at 4°C. The supernatants were subjected to sodium dodecyl sulfate polyacrylamide gel electrophoresis (SDS-PAGE), followed by immunoblotting with horseradish peroxidase (HRP)-conjugated anti-phosphorylated tyrosine antibody (clone pY20, GE Healthcare,). Chemiluminescence was detected using Super Signal West Femto Chemiluminescent Substrate (Pierce) and the ImageQuant LAS 4000 mini digital imaging system (GE Healthcare).

For the measurement of the activation of Jurkat/FcγR/NFAT-Luc cells, Jurkat/FcγR/NFAT-Luc cells were crosslinked by anti-CD32 or anti-CD16 antibody as described above, and incubated at 37°C in 5% CO_2_. At each time point, the luciferase activities were measured by using ONE-Glo Luciferase Assay System (Promega) and the EnSpire Multimode Plate Reader (PerkinElmer).

### Antibodies

DNA fragments encoding the rituximab heavy-chain and light-chain variable domains were synthesized by Integrated DNA Technologies, and subcloned into pFUSE-CHIg-hG1 and pFUSE2-CLIg-hk vector (InvivoGen), respectively. The expression vectors encoding the rituximab Fc variants with G236A/S239D/I332E or L234A/L235A substitutions were constructed by using synthesized DNA fragments. Rituximab and its Fc variants were expressed using the FreeStyle MAX CHO Expression System (Life Technologies).Briefly, CHO-S cells were co-transfected with the plasmid vectors expressing rituximab heavy chain and light chain by using the FreeStyle MAX Reagent, and they were then cultured for 6 days in FreeStyle CHO Expression Medium. The cell culture supernatant was collected by centrifugation (400 g for 10 min) and applied to a HiTrap Protein G HP column (GE Healthcare) equilibrated with 20 mM phosphate buffer (pH 6.8). After the column was washed with 20 mM phosphate buffer (pH 6.8), mAbs were eluted by 0.1 M Glycine-HCl (pH 3.0) and neutralized by 1 M Tris-HCl (pH 8.0), followed by desalting using a PD-10 column (GE Healthcare) equilibrated with phosphate-buffered saline (PBS). The concentration of purified mAb was determined by spectrophotometry using the NanoDrop 2000c spectrophotometer (Thermo Scientific). Cetuximab (Erbitux, Merck Serono) and panitumumab (Vectibix, Amgen) were purchased via reagent distributors.

### ADCC Assay

Cryopreserved human PBMCs were obtained from Cellular Technology Limited, and thawed just before ADCC assay according to the manufacturer’s protocol. Daudi (1×10^4^ cells/well) and human PBMC (2×10^5^ cells/well) suspended in CTL-Test Medium (Cellular Technology Limited) were seeded in a 96-well plate with serially diluted rituximab. After incubation for 4 hr at 37°C in 5% CO_2_, lactate dehydrogenase (LDH) activity of cell culture supernatants were measured by using Cytotoxicity Detection Kit^PLUS^ (LDH) (Roche Applied Science). The percentage cytotoxicity was calculated as described in the manufacturer’s protocol. The ethical review boards of the National Institute of Health Sciences approved the use of human PBMC in this study.

### FcγR Reporter Assay

Daudi (2×10^4^ cells/well) and Jurkat/FcγRs/NFAT-Luc (1×10^5^ cells/well) suspended in Opti-MEM I Reduced Serum Media were seeded in a 96-well plate with serially diluted rituximab. After incubation for 5 hr at 37°C in 5% CO_2_, we measured the luciferase activities by using ONE-Glo Luciferase Assay System (Promega) and the EnSpire Multimode Plate Reader (PerkinElmer).

For the assays using anti-EGFR mAbs, A431 (2×10^4^ cells/well) were seeded in a 96-well plate and cultured for 24 hr at 37°C in 5% CO_2_. After the medium was removed, Jurkat/FcγRs/NFAT-Luc (1×10^5^ cells/well) suspended in Opti-MEM I Reduced Serum Media were added with serially diluted anti-EGFR mAbs and incubated for 5 hr, followed by measurement of the luciferase activity as described above.

### Forced Oxidization of Cetuximab by t-BHP

Cetuximab (5 mg/mL) was incubated in PBS containing 0.2, 1 or 5% of tert-Butyl hydroperoxide (t-BHP) (Wako) for 3 hr at 37°C. After the incubation, t-BHP was removed from the reaction mixtures by applying the mixtures two times to a PD Spin Trap G-25 column (GE Healthcare) equilibrated with PBS.

### SPR Analysis

A Biacore T200 SPR biosensor (GE Healthcare) and CM5 sensor chip were used to evaluate the binding properties of mAbs. All measurements were performed at 25°C, with a flow rate of 30 µL/min for analytes. The binding affinity of mAbs with FcRn was measured as described.[29] Briefly, the recombinant human FcRn was immobilized onto a sensor chip (GE Healthcare) by the amine coupling method (∼350 RU). The mAb samples in twofold serial dilutions from 670 to 42 nM with the running buffer (50 mM sodium phosphate/150 mM NaCl [pH 6.0]) were injected for 120 sec followed by a 150-sec dissociation phase.

The surface was regenerated by injecting the buffer of 100 mM Tris with 200 mM NaCl [pH 8.0] for 30 sec. The dissociation constant (*K*_D_) was calculated from the sensorgrams using the bivalent analyte model and setting the bulk reflective index to zero using BICORE T200 evaluation software (GE Healthcare). We measured the affinities of mAbs with human FcγRIIa and FcγRIIIa using the sensor chip with which the recombinant ectodomains of human FcγRIIa or FcγRIIIa (Sino Biological) were immobilized by the amine coupling method.

mAb samples in twofold serial dilutions with HBS-EP buffer (50 mM sodium phosphate/150 mM NaCl [pH 6.0]) were injected for 3 min followed by a 4-min dissociation phase. The analyte concentrations were from 2680 to 21 nM and from 335 to 21 nM for FcγRIIa and FcγRIIIa, respectively. The surface was regenerated by injecting 10 mM NaOH for 30 sec. The dissociation constant *K*_D_ for FcγRIIa was calculated by the steady-state method. The dissociation constant *K*_D_ for FcγRIIIa was calculated by the two-state model[30].

For the measurement of the binding affinity with EGFR, anti-human IgG antibody (GE Healthcare) was immobilized onto sample and reference flow cells of a sensor chip. A mAb sample (0.2 µg/mL) was captured on a sensorchip by injecting it into the sample flow cell at a flow rate of 10 µL/min for 1 min. The recombinant ectodomain of human EGF receptor (Sino Biological) in twofold serial dilutions from 40 to 2.5 nM with HBS-EP buffer was injected for 5 min followed by a 10-min dissociation phase. After each cycle, the surface was regenerated by injecting 3 M MgCl_2_ for 30 sec. The dissociation constant *K*_D_ for EGFR was calculated using the 1∶1 binding model. In all experiments, each binding sensorgram from the sample flow cell was corrected for both the surface blank and the buffer injection control (double reference).[31] Statistical analysis was performed using one-way analysis of variance (ANOVA) with Tukey’s multiple comparison test (PRISM version 5.02; Graphpad Software).

## Results

### Establishment of FcγR-expressing Reporter Cell Lines

**Figure 1:**
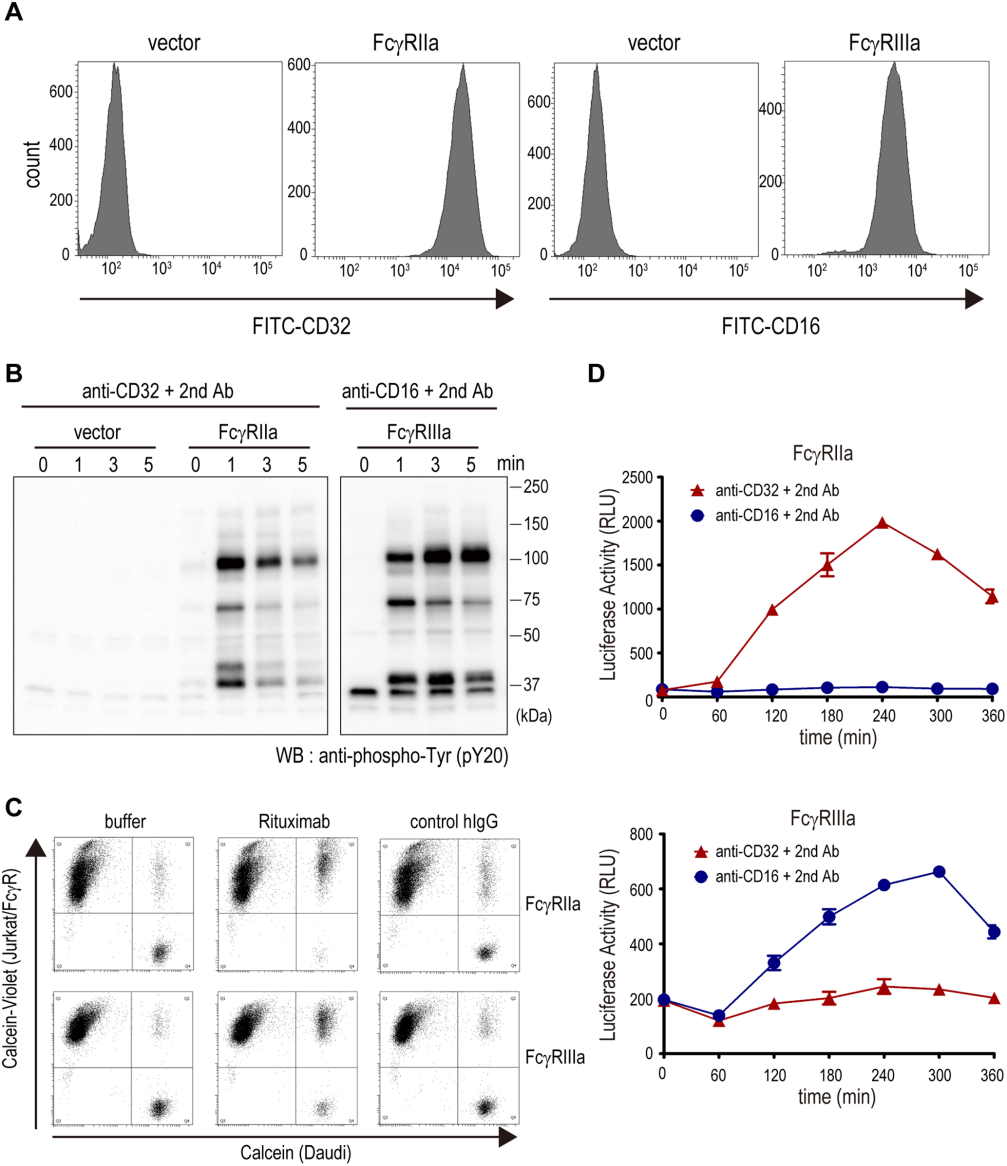
Establishment of the FcγR-expressing reporter cell lines. (A) The cell surface expressions of FcγRs in Jurkat/FcγR cells were analyzed by flow cytometric analysis. (B) The crosslinking of FcγRs by anti-FcγR monoclonal antibodies induced the tyrosine-phosphorylated proteins in Jurkat/FcγR cells. (C) Bridging between calcein-labeled Daudi and calcein-violet-labeled Jurkat/FcγR cells via rituximab was analyzed by flow cytometry. (D) The crosslinking of FcγRs by anti-FcγR monoclonal antibodies induced the luciferase activities in Jurkat/FcγR/NFAT-Luc cells. The assays were performed in triplicate, and the data are the mean ± SEM.

To develop the reporter cell lines measuring FcγRs activation, we first generated the Jurkat cell line which stably expresses FcγRIIa (Jurkat/FcγRIIa). The cell line expressing FcγRIIIa (Jurkat/FcγRIIIa) was also established. The specific expression of FcγR in each cell line was confirmed by a flow cytometric analysis (Fig. 1A). We next examined whether these cell lines were activated via the crosslinking of FcγRs. The crosslinking of FcγRs by immune complexes induces the phosphorylation of immunoreceptor tyrosine-based activation motif (ITAM) located on the cytoplasmic tail of the FcγRs, which triggers the activation of a downstream signaling pathway[17].

As shown in Figure 1B, the crosslinking of FcγRs by anti-FcγRs antibodies increased the tyrosine-phosphorylated proteins in the Jurkat/FcγR cells, indicating that both Jurkat/FcγRIIa and Jurkat/FcγRIIIa cells are responsive to FcγR stimulation. To exert ADCC, FcγRs-expressing effector cells recognize the mAbs bound to antigen on the surface of target cells. This bridging of target and effector cells by the mAbs is a critical step for the initiation of ADCC. To assess the bridging ability of Jurkat/FcγR cells, we performed the flow cytometry-based bridging assay. Calcein-labeled Daudi cells, a human Burkitt’s lymphoma cell line that strongly expresses CD20, and calcein-violet-labeled Jurkat/FcγR cells were co-incubated with or without an anti-CD20 mAb (rituximab) and analyzed by flow cytometry.

With the addition of rituximab, the calcein/calcein-violet double-positive population was significantly increased (Fig. 1C), indicating that the Jurkat/FcγR cells recognized rituximab bound to CD20 at the surface of the Daudi cells; that is, Daudi and Jurkat/FcγR cells were bridged by rituximab. These results suggest that the Jurkat/FcγRIIa and Jurkat/FcγRIIIa cell lines we developed can function as reporter cells mimicking FcγR-expressing immune cells such as NK cells and macrophages.

To monitor the activation of Jurkat/FcγR cells conveniently, we introduced the luciferase reporter gene driven by NFAT-response element (NFAT-RE) into Jurkat/FcγR cells and established the reporter cell lines Jurkat/FcγRIIa/NFAT-Luc and Jurkat/FcγRIIIa/NFAT-Luc. NFAT is a well-known transcription factor activated by intracellular calcium signaling, and the reporter gene driven by NFAT-RE has been known as a useful tool for monitoring FcγR activation.[21] We confirmed that the luciferase reporter was activated via the crosslinking of FcγRs by anti-FcγRs antibodies (Fig. 1D).

### Measurement of mAb-dependent FcγRIIa Activation by Using Jurkat/FcγRIIa/NFAT-Luc

**Figure 2:**
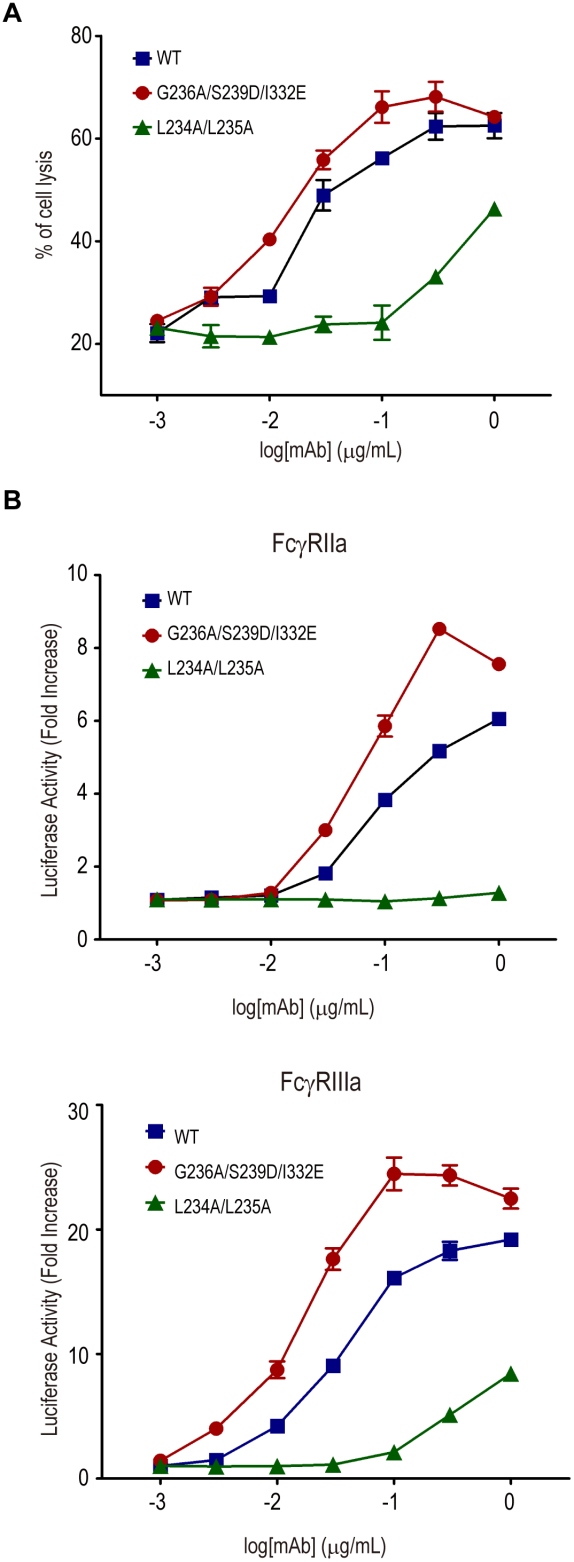
Activation of Jurkat/FcγR/NFAT-Luc cells by CD20-bound rituximab. (A) Daudi and human PBMC were co-cultured (the effector to target cell ratio was 20∶l) in the presence of serially diluted rituximab or its Fc-engineered variants: G236A/S239D/I332E with higher FcγR binding and L234A/L235A with lower FcγR binding. Percentage cytotoxicity calculated by LDH activity released from damaged cells is represented on the graphs. The assays were performed in triplicate, and the data are the mean ± SEM. The rituximab G236A/S239D/I332E variant exhibited ADCC activity (EC_50_ = 0.013 µg/ml) more strongly than wild-type rituximab (EC_50_ = 0.023 µg/ml) (p<0.05, Graphpad Prism Software). (B) Daudi and Jurkat/FcγR/NFAT-Luc cells were co-cultured in the presence of serially diluted rituximab or its Fc-engineered variants. Luciferase activity (i.e., the fold increase compared to the control sample without mAbs) is represented on the graphs. The assays were performed in triplicate, and the data are the mean ± SEM. Jurkat/FcγRIIa/NFAT-Luc cells were activated by the rituximab G236A/S239D/I332E variant (EC_50_ = 0.057 µg/ml) more strongly than wild-type rituximab (EC_50_ = 0.094 µg/ml) (p<0.005, Graphpad Prism Software). Jurkat/FcγRIIIa/NFAT-Luc cells were activated by the rituximab G236A/S239D/I332E variant (EC_50_ = 0.016 µg/ml) more strongly than wild-type rituximab (EC_50_ = 0.034 µg/ml) (p<0.0001, Graphpad Prism Software).

To assess the usefulness of Jurkat/FcγRIIa/NFAT-Luc cells for the measurement of mAb-dependent FcγRIIa activation, we first performed the assay using anti-CD20 mAb rituximab and its engineered Fc variants with increased or decreased affinities to FcγRs. The engineered Fc variant with G236A/S239D/I332E substitutions has been reported to exhibit higher FcγRIIa and FcγRIIIa binding and enhanced activation of effector cells, including NK cells and macrophages.[6] On the other hand, the variant with L234A/L235A substitutions is known to bind FcγRs weakly and activate effector cells with lower efficiency.[32] We confirmed that the rituximab G236A/S239D/I332E variant exhibit ADCC activity more strongly than wild-type rituximab, whereas the rituximab L234A/L235A variant exhibit lower ADCC activity than wild-type rituximab (Fig. 2A).

We found that the luciferase activity of Jurkat/FcγRIIa/NFAT-Luc cells as well as Jurkat/FcγRIIIa/NFAT-Luc cells was dose-dependently increased by rituximab in co-culture with Daudi cells (Fig. 2B), suggesting that the activation of FcγRIIa by the bridging of target and effector cells via mAbs can be assessed by using Jurkat/FcγRIIa/NFAT-Luc cells. Furthermore, the Jurkat/FcγRIIa/NFAT-Luc cells were activated by the rituximab G236A/S239D/I332E variant more strongly than wild-type rituximab, whereas they were hardly activated by the rituximab L234A/L235A variant. Similar results were observed by using Jurkat/FcγRIIIa/NFAT-Luc cells (Fig. 2B).

We next performed the assay using A431 cells (a human epithelial carcinoma cell line that overexpresses EGFR) as target cells and two anti-EGFR mAbs, cetuximab and panitumumab. Cetuximab is a human-mouse chimeric IgG1 mAb, and panitumumab is a fully human IgG2 mAb. Regarding the characteristics of the human IgG subclass, IgG1 binds FcγRIIIa and exhibits ADCC efficiently by NK cells, whereas IgG2 shows weak binding activity to FcγRIIIa.

**Figure 3:**
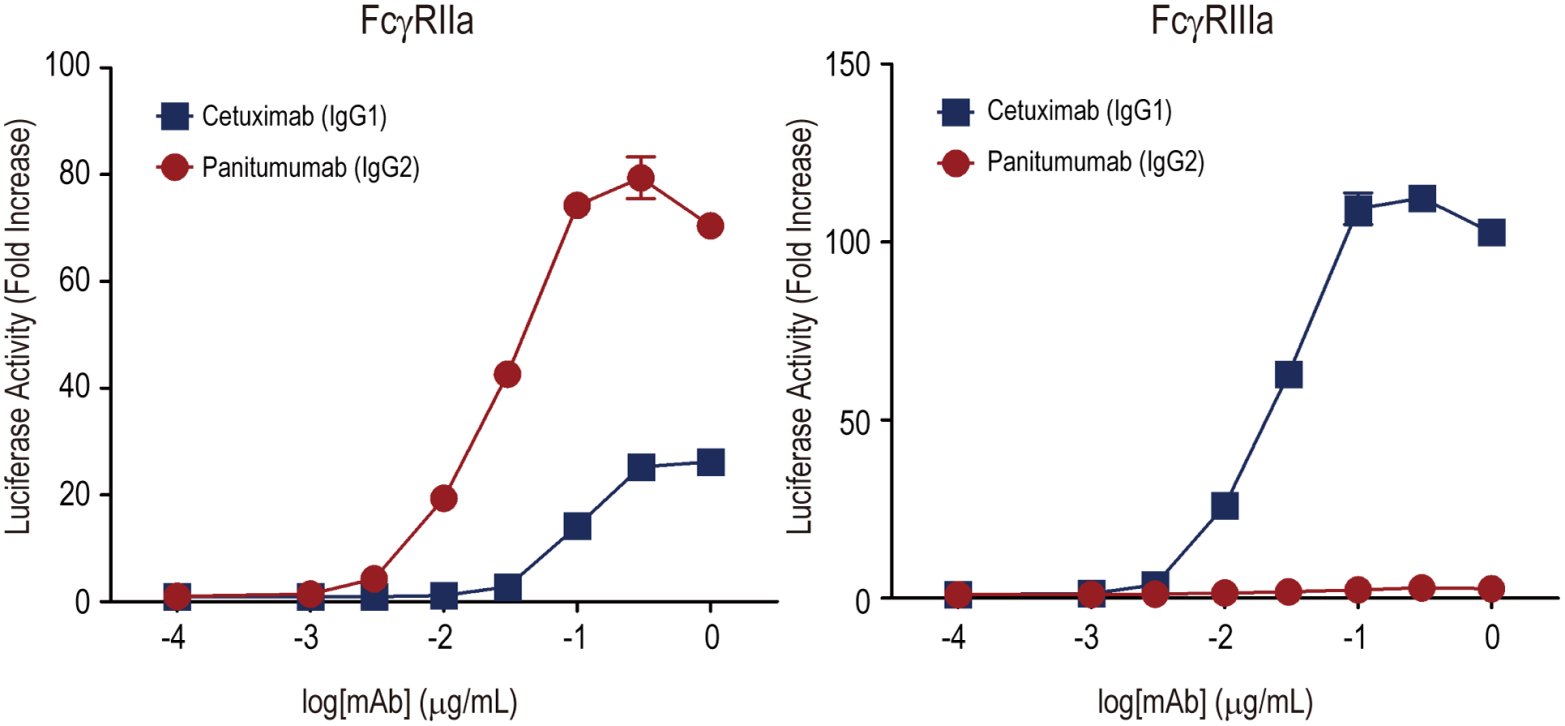
Activation of Jurkat/FcγR/NFAT-Luc cells by EGFR-bound cetuximab and panitumumab. A431 and Jurkat/FcγR/NFAT-Luc cells were co-cultured in the presence of serially diluted cetuximab (IgG1 subclass) or panitumumab (IgG2 subclass). The luciferase activity (i.e., the fold increase compared to the control sample without mAbs) is represented on the graphs. The assays were performed in triplicate, and the data are the mean ± SEM. Panitumumab activated Jurkat/FcγRIIa/NFAT-Luc cells more strongly than cetuximab (p<0.0001, Graphpad Prism Software).

Panitumumab has been thought to be inactive regarding the induction of ADCC. However, Schneider-Merck and coworkers reported that panitumumab exerted ADCC by myeloid effector cells (i.e., neutrophils and monocytes) isolated from human blood, and that the cytotoxicity may be triggered by FcγRIIa.[27] As shown in Figure 3, cetuximab (IgG1) activated the Jurkat/FcγRIIIa/NFAT-Luc cells effectively in a dose-dependent manner, whereas panitumumab (IgG2) did not. Both cetuximab and panitumumab activated Jurkat/FcγRIIa/NFAT-Luc cells, and the activity of panitumumab was significantly higher than that of cetuximab. These results were consistent with those of the previous study [27] using primary cultured effector cells, suggesting that the Jurkat/FcγRIIa/NFAT-Luc cell lines are useful for estimating the potency of IgG2 mAbs to activate FcγRIIa-expressing effector cells.

### Impact of Oxidation on the Ability of Cetuximab to Activate FcγRs

To estimate the usefulness of Jurkat/FcγRIIa/NFAT-Luc and Jurkat/FcγRIIIa/NFAT-Luc cells in mAbs-variant characterization, we analyzed the impact of methionine oxidation on the ability of cetuximab to activate FcγRs. Methionine oxidation in IgG Fc is known to decrease the affinity of IgG for FcRn, an Fc receptor regulating IgG recycling in endothelial and blood cells, which leads to reduce the serum half-life of IgG.[33]–[35] However, the effects of methionine oxidation in IgG Fc on FcγR activation are not fully understood.

**Figure 4:**
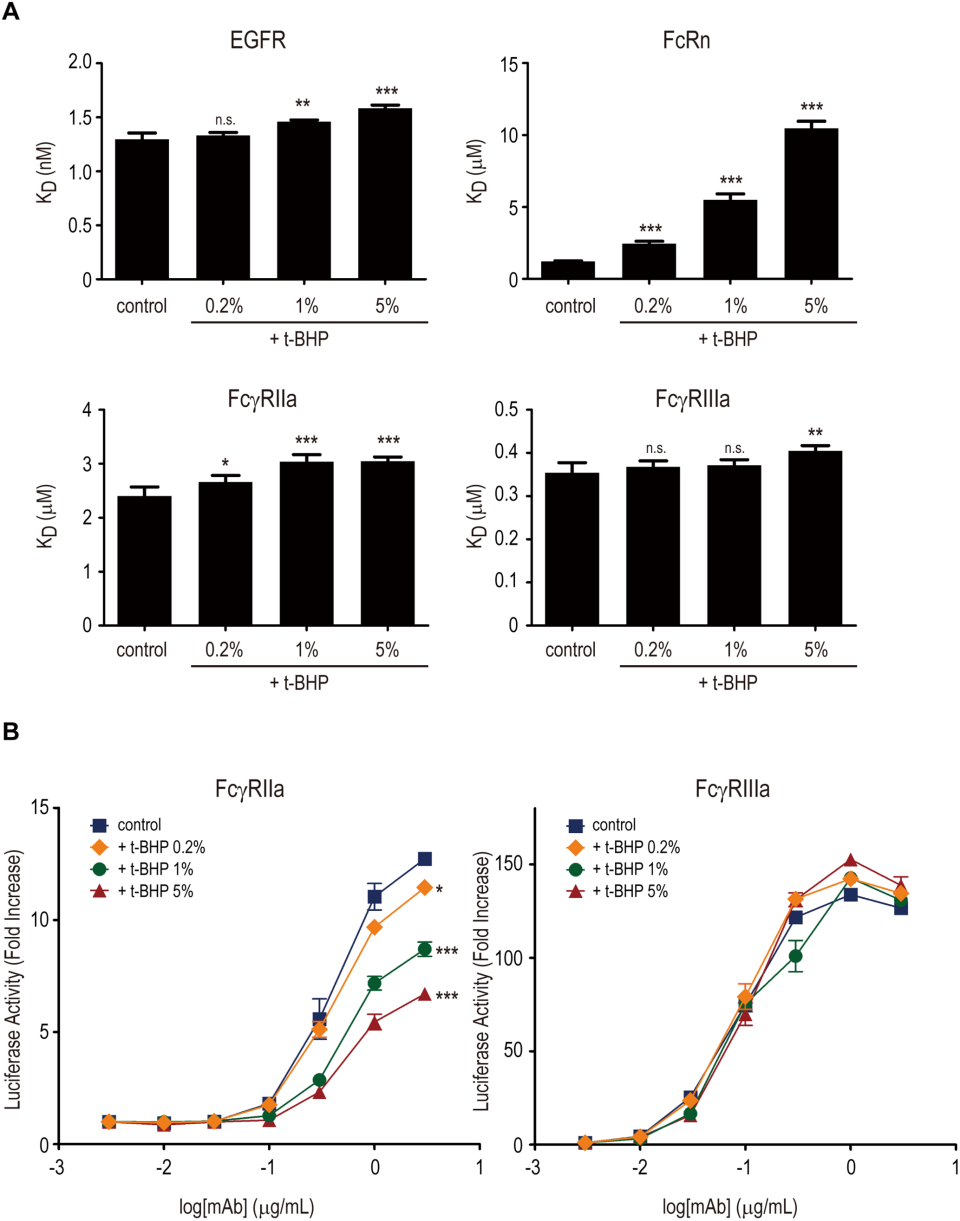
Effects of methionine oxidation on the activation of FcγRIIa and FcγRIIIa by cetuximab. (A) Binding affinities of t-BHP-treated cetuximab to EGFR, FcRn, FcγRIIa and FcγRIIIa were measured by an SPR analysis. Dissociation constant (K_D_) values are represented as mean ± SD (n = 3). *, p<0.05; **, p<0.01; ***, p<0.005; n.s, not significant. (B) Activation of FcγRIIa and FcγRIIIa by t-BHP-treated cetuximab was measured by an FcγR reporter assay using A431 cells as target cells. Methionine oxidation caused by t-BHP treatment dose-dependently decreased the activation of FcγRIIa (*, p<0.05; ***, p<0.005), but not that of FcγRIIIa (not significant).

To prepare the samples with different levels of oxidation, cetuximab was treated with tert-butyl hydroperoxide (t-BHP), which is known to oxidate methionine residues preferentially in proteins, at different concentrations. Surface plasmon resonance (SPR) analysis using these oxidized mAbs showed that the binding affinity of cetuximab for FcRn was decreased in a dose-dependent manner by t-BHP (Fig. 4A), suggesting that t-BHP treatment dose-dependently induced methionine oxidation in Fc region of cetuximab.

On the other hand, t-BHP treatment induced only a slight decrease in the binding activities of cetuximab for EGFR, FcγRIIa and FcγRIIIa (Fig. 4A). We next performed the cell-based reporter assay using Jurkat/FcγRIIa/NFAT-Luc and Jurkat/FcγRIIIa/NFAT-Luc cells to estimate the effect of oxidation in cetuximab Fc on antigen-binding-dependent FcγR activation. In contrast to the *in vitro* binding analysis using SPR, we found that the t-BHP treatment significantly diminished the FcγRIIa activation by EGFR-bound cetuximab, although FcγRIIIa activation was not influenced by t-BHP treatment (Fig. 4B). These results suggest that methionine oxidation may decrease the FcγRIIa activation by EGFR-bound cetuximab and that Jurkat/FcγRIIa/NFAT-Luc cells are useful for monitoring the changes of mAb biological activities.

## Discussion

Cell-based assays reflecting mechanisms of action are indispensable for assessing the biological activities of therapeutic mAbs from the early stage of drug discovery to post-approval quality control tests. In association with the progress in the development of tumor-targeting mAbs and their engineered variants with higher ADCC activity, various methods of measuring ADCC have been developed.[20], [21], [36]–[38] However, most of these assay methods were designed to estimate ADCC mediated by NK cells via FcγRIIIa, and the contribution of FcγRIIa was hardly detected.

Considering the importance of FcγRIIa-mediated cytotoxicity by mAbs, in the present study we developed a cell-based assay using Jurkat/FcγRIIa/NFAT-Luc reporter cells. The FcγRIIa-reporter assay can measure the activation of FcγRIIa by antigen-bound mAbs, and is a promising tool for estimating the tumor-targeting mAbs and their Fc-engineered variants with enhanced or decreased FcγRIIa-binding affinity.

During the development of therapeutic mAbs, IgG subclass selection and the evaluation of their interactions with FcγRs are important issues. Among the human IgG subclasses, IgG1 is the most commonly used subclass for therapeutic mAbs. In particular, the effector functions of IgG1 (i.e., the induction of ADCC and complement-dependent cytotoxicity) are important for the mAbs inducing tumor-cell killing, and Fc engineering technologies enhancing the effector functions of IgG1 have been applied to the development of novel anti-cancer therapeutic mAbs.

However, immune reactions mediated by complement or FcγR activation can sometimes be responsible for the adverse reactions by unwanted cytotoxicity to antigen-expressing cells or unwanted activation of effector cells. Therefore, when target-cell killing via effector function is not required, IgG4 is a preferable subclass to reduce the unwanted immune reactions, because IgG4 induces effector functions more weakly than IgG1. IgG2 has also been thought to be the isotype of choice for mAbs not requiring immune reactions because of its inability to activate the classical complement pathway and bind to activating FcγRs (FcγRI and FcγRIIIa).[23], [39] However, IgG2 binds FcγRIIa, which is widely expressed in myeloid effector cells including monocytes, macrophages and neutrophils, and IgG2 immune complexes activate these effector cells via FcγRIIa crosslinking.

The activation of FcγRIIa is thought to be involved not only in the efficacy but also in the safety of therapeutic mAbs. Therapeutic mAbs sometimes induce infusion and hypersensitivity reactions, which are generic terms for acute adverse reactions that can be caused by an immunologic mechanism, including cytokine-release syndrome (CRS), allergic reactions, and pseudoallergic reactions.[40], [41] There are some putative molecular mechanisms in which mAbs cause the release of inflammatory cytokines. mAbs targeting the cell-surface antigens expressed in immune cells may induce the cytokine release from the target cells by agonistic activity (e.g., muromonab-CD3 [42] and TGN1412 [43]). Therapeutic mAbs also have the potential to trigger cytokine release by an interaction with activating FcγRs on immune effector cells, regardless of whether or not target cell killing is required for their pharmacological activities. The involvement of FcγRIIa in cytokine release and the adverse effects of murine anti-CD3 antibody were reported by Tax et al.[44] In addition, the contribution of FcγRIIa to IgG-induced allergic reactions and anaphylaxis was recently reported by Jönsson et al.[45], [46] Those researchers found that FcγRIIa was sufficient to trigger active and passive anaphylaxis in an FcγRIIa-transgenic mice model, and human mast cells, monocytes and neutrophils produced an anaphylactogenic mediator by FcγRIIa crosslinking.

These findings suggest the importance of activating FcγRs in the pathogenic mechanisms of infusion and hypersensitivity reactions by therapeutic mAbs. Thus, when considering the safety profiles of therapeutic mAbs, it is necessary to evaluate the activation of FcγRs by the mAbs. As described above, FcγRIIa is expressed widely in myeloid effector cells including neutrophils, and it is thought to play a pivotal role in the activation of these cells. Our FcγRIIa-reporter assay can measure the activation of FcγRIIa by antigen-bound mAbs *in vitro*, and may contribute to the assessment of the safety profile of therapeutic mAbs.

In the present study, we also showed the usefulness of the FcγR-reporter assay for the characterization of mAb variants. Oxidation is one of the most common modifications that occur during the manufacturing and storage processes of therapeutic proteins.[47], [48] Among the various amino acid residues which can be oxidized, methionine is the most sensitive residue to oxidation. Human IgG1 contains two conserved methionine residues (Met-252 and Met-428) in its constant region. Previous studies revealed that methionine oxidation induces structural changes in the IgG Fc region,[49] decreases the binding affinity to FcRn,[33], [34] and reduces the serum half-life of IgG[43].

However, the effects of methionine oxidation on the ability of mAbs to activate FcγRs have not been fully understood, although a subtle decrease in FcγRIIa binding was observed in methionine-oxidized trastuzumab.[33] We reported here that oxidation in cetuximab significantly decreased the EGFR binding-dependent activation of FcγRIIa-expressing reporter cells, but not that of FcγRIIIa-expressing reporter cells. Interestingly, oxidation induced only a slight change in the binding affinities to EGFR and FcγRIIa in an SPR analysis using recombinant proteins.

There are two potential mechanisms to explain the difference between the results of the *in vitro* binding assay and those of the cell-based assay: first, that the slight decrease in the binding affinity to both EGFR and FcγRIIa observed in the SPR analysis may have synergistically affected the FcγRIIa-activation in the cell-based assay; and second, that oxidation may influence the structural changes at the Fc region caused by antigen binding, resulting in the reduced efficacy of EGFR-bound cetuximab to activate FcγRIIa. In both cases, the cell-based assay mimicking the *in vivo* situation is thought to be superior to the *in vitro* binding assay using recombinant proteins when characterizing the biological activities of mAb variants. To our knowledge, this is the first report demonstrating the effect of methionine oxidation on the efficacy of mAbs to activate FcγRs. Further studies are required to reveal the detailed mechanisms underlying the effects of methionine oxidation on the structure of mAbs’ Fc region and its functions.

In conclusion, we reported here the development of a cell-based assay using an FcγRIIa-expressing reporter cell line. Our FcγRIIa reporter cell assay can evaluate the activation of FcγRIIa by antigen-bound mAbs *in vitro* and is useful for the characterization of therapeutic mAbs, including Fc-engineered mAbs, IgG2-subclass mAbs, and their product-related variants. Although further studies are required to reveal a correlation between *in vivo* efficacy and *in vitro* FcγRIIa activation, as is true of the previously reported FcγRIIIa reporter cell assay, our FcγRIIa reporter cell assay is a promising tool for the characterization of therapeutic mAbs.
